# Effect of Fruit and Vegetable Consumption on Human Health: An Update of the Literature

**DOI:** 10.3390/foods13193149

**Published:** 2024-10-02

**Authors:** Chiara Devirgiliis, Emilia Guberti, Lorenza Mistura, Antonio Raffo

**Affiliations:** 1 Research Centre for Food and Nutrition, CREA (Consiglio per la Ricerca in Agricoltura e l’Analisi Dell’Economia Agraria), Via Ardeatina 546, 00178 Rome, Italy; chiara.devirgiliis@crea.gov.it (C.D.); lorenza.mistura@crea.gov.it (L.M.); 2 The “Food and Nutrition” Working Group of the Italian Society of Hygiene, Preventive Medicine and Public Health (SItI-Società Italiana di Igiene Medicina Preventiva e Sanità Pubblica), Viale Città d’Europa 74, 00144 Rome, Italy; emilia.guberti@gmail.com

**Keywords:** disease prevention, fiber-rich foods, plant-based foods, healthy diet, plant-based diet, nutrition guidelines

## Abstract

Several meta-analyses have consistently demonstrated that the consumption of an adequate level of fruit and vegetables (F&V), along with other food groups, is associated with a low risk of all-cause mortality, and, as such, represents one of the major modifiable risk factors related to the growing burden of Non-Communicable Diseases (NCDs). The aim of the present narrative review was to provide an up-to-date analysis of systematic reviews and meta-analyses published in the past five years, dealing with the effects of F&V consumption on human health, focusing on specific pathologies, such as total mortality, cancer, cardiovascular diseases (CVDs), type 2 diabetes, intestinal inflammation, and bone and respiratory illnesses. The results of our evaluation confirmed and consolidated the protective role of F&V consumption against the development of NCDs, especially CVDs. However, the need to corroborate existing evidence and clarify the role of confounding factors by performing additional randomized control trials and adopting more standardized approaches and study designs also emerged. Moreover, evaluating the protective role of fruit and vegetables as separate food categories appeared to be one of the most interesting areas to investigate in the near future. Overall, these outcomes could help in addressing future research to better establish a causal relationship between F&V consumption and human health.

## 1. Introduction

As constantly recommended by the World Health Organization (WHO), the consumption of adequate levels of fruit and vegetables (F&V) represents one of the fundamental elements of a healthy diet. In line with the latest WHO statement on a healthy diet, for an adult population, the consumption of at least 400 g, or five servings, of F&V per day reduces the risk of Non-Communicable Diseases (NCDs) and contributes to ensuring a proper daily intake of dietary fiber [1]. An inadequate level of F&V consumption is indeed considered one of the major modifiable risk factors contributing to the growing burden of NCDs [2].

Numerous meta-analyses have shown how achieving the recommended levels of F&V consumption, together with other foods (fish, nuts, and whole grains), represents one of the most important dietary factors associated with a low risk of all-cause mortality [3]. Globally, this evidence has been confirmed by the results of the Global Burden of Disease (GBD) study carried out on data available up to 2017, which highlighted, conversely, how diets low in F&V represent one of the main dietary risk factors [4]. Considering, in particular, the European Union area consisting of 28 countries, it has been estimated that approximately 172,000 and 118,000 premature deaths can be attributed to diets poor in F&V, respectively. Furthermore, based on the results of the analysis of Disability Adjusted Life Years (DALYs), conducted as part of the same study, it was estimated that approximately 3.4 and 1.8 million DALYs can be attributed to diets poor in F&V, respectively [4].

The global consensus on the above-mentioned evidence is demonstrated by the recommendation to increase F&V consumption in the dietary guidelines of over 100 countries worldwide. These guidelines take into account specific local conditions, such as the nutritional status of the population, food availability, culinary culture, and dietary habits in different countries [3,5]. Particularly, the specific quantitative recommendation of consuming 400 g, or five servings, is present in the guidelines of about one-third of the countries, while in half of the cases, a similar quantitative indication is conveyed through key messages or food advice [5].

In particular, several guidelines recommend increasing F&V consumption because these foods are characterized by low energy density, provide dietary fiber, supply essential vitamins and minerals, and contain bioactive compounds with protective effects on human health. As mentioned above, the role of F&V in the diet goes beyond providing essential nutrients and includes preventive action against various NCDs.

Moreover, it is noteworthy that with the recent publication of the new guidelines on carbohydrate intake in adults and children, WHO has provided, for the first time, indications on recommended levels of F&V consumption in children and adolescents, specifying a level of 250 g/day for children aged 2–5 years, 350 g for the age group 6–9 years, and 400 g representing the recommended level for adults for the age group ≥ 10 years [1]. However, differently from what is stated for the adult population, this second set of recommendations is classified as conditional instead of strong. This means that according to the WHO, it is less certain that the desirable outcomes of implementing the recommendation outweigh the unwanted effects, or that the expected benefits are modest. Therefore, a deep discussion among policymakers would be needed before adopting a conditional recommendation as a policy.

In any case, despite the recommendation to consume larger quantities of F&V daily, consumption levels remain inadequate in many countries all over the world [6]. In Europe, based on official statistics from EUROSTAT and FAOSTAT, the average F&V consumption at the population level dropped to 350 g/day/capita in 2022, corresponding to a 5% reduction from 2021 and an approximately 3% decline when compared to the average level of the previous 5 years. The observed amount of 350 g/day/capita was, however, 12% lower than the 400 g/day/capita recommended by the WHO [7]. Moreover, as few as six EU countries reached the goal of 400 g of F&V daily consumption. When considering the number of F&V portions consumed daily, at the European level, EUROSTAT estimated that, on average, only 12% of the adult population achieves the goal of at least five servings [8]. In addition, another source of data is the EFSA Comprehensive European Food Consumption Database, which provided the mean intake of F&V for the adult population (18–64 years) of 321.6 g/day considering the twenty-one countries that carried out the food consumption survey over the past 10 years [9].

Several strategies have been proposed to promote an increase in the consumption of F&V. Among these, behavioral strategies aimed at enhancing the capacity of individuals to improve their diet, as well as innovation in F&V processing and supply chains aimed at increasing the availability of fresh products on the market and the opportunities for consuming F&V, are considered particularly important [3,10]. From this perspective, research activities aimed at improving the quality, shelf-life, and safety of fresh high-convenience foods can increase options for F&V consumption [11].

The establishment of proper recommendations relies on scientific evidence based on clinical studies and epidemiological evidence linking F&V intake to health promotion and chronic disease prevention, in order to confirm the current knowledge or to establish new dietary guidelines.

The aim of this narrative review was therefore to provide an up-to-date analysis of the recent scientific evidence related to the effects of F&V consumption on human health, focusing on specific groups of pathologies. In particular, the core research purpose was to select and analyze systematic reviews and meta-analyses published in the past 5 years to highlight the most robust and convincing health benefits emerging from intervention and observational studies, but also to point out existing concerns and gaps, that could aid in addressing future research to better establish a causal relationship between F&V consumption and human health.

## 2. Literature Search and Selection Criteria

A literature search was conducted by querying the PubMed database using the following search string:

((“fruit”[MeSH Terms] OR “fruit”[All Fields] OR “fruits”[All Fields] OR “fruit s”[All Fields] OR “fruited”[All Fields] OR “fruiting”[All Fields]) AND (“vegetables”[MeSH Terms] OR “vegetables”[All Fields] OR “vegetable”[All Fields]) AND (“intake”[All Fields] OR “intake s”[All Fields] OR “intakes”[All Fields]) AND (“health”[MeSH Terms] OR “health”[All Fields] OR “health s”[All Fields] OR “healthful”[All Fields] OR “healthfulness”[All Fields] OR “healths”[All Fields])), and limiting the time span to the past 5 years. The filters “Meta-Analysis” and “Systematic Review” were applied to restrict the search to systematic reviews and meta-analyses.

The screening process yielded 268 publications, which were evaluated, by reading the abstract, using as inclusion criteria: works written in English and with an available abstract; works that reported a clear reference to the effects of F&V consumption on human health; works focused on the following types of pathologies: non-communicable diseases, namely obesity, cancer and cardiovascular pathologies, total mortality, and other pathologies such as type 2 diabetes, intestinal inflammation, and bone and respiratory diseases. Concerning the exclusion criteria, those works that only took into consideration strategies aimed at increasing F&V consumption, reported only consumption data, or analyzed the effects of individual F&V components were excluded from the analysis.

The subsequent paragraphs describe in detail the effects of F&V consumption on human health in relation to the categories of pathologies indicated previously, based on the analysis of the recent literature conducted on the 28 publications selected following the evaluation (Table 1).

## 3. Effects of Fruit and Vegetable Consumption on All-Cause Mortality

All-cause mortality is defined as the death rate from all causes of death for a population in a given time period. Over the past 50 years, several lifestyle factors have been identified as modifiable determinants associated with premature death. Even in the absence of an understanding of the biological mechanisms responsible for these associations, the identification of epidemiological risk factors is considered useful in order to modify the probability of death and monitor the state of public health. From this perspective, a lot of studies have highlighted the role of the consumption of adequate levels of F&V in reducing the risk of all-cause mortality [3]. The above-reported findings mainly derived from the GBD study and have been confirmed in other more recent works carried out in different geographical areas [40,41].

The six meta-analyses and systematic reviews published in the 2018–2023 time frame, selected from the research conducted in the present review and focused on all-cause mortality (Table 1), are described and discussed in detail below.

The results of two prospective cohort studies (66,719 women from the Nurses’ Health Study and 42,016 men from the Health Professionals Follow-Up Study) in the United States and a meta-analysis of 26 cohort studies, carried out in different countries on distinct continents (comprising almost two million participants), have robustly highlighted the association between a greater intake of F&V with a lower risk of total and cause-specific mortality (cancer, cardiovascular diseases, and respiratory diseases) (*P_nonlinear_* < 0.001). The lowest risk of mortality was associated with about five servings of F&V daily intake when compared with the reference intake, i.e., two servings/d (RR 0.87; 95% CI: 0.85–0.88), while above this level, the risk was not further decreased. The minimum intake value to guarantee the reduction in mortality risk was two portions per day for fruit intake and three portions per day for vegetable intake, with the exclusion of fruit juices and potatoes. These results corroborate the recommendation to increase F&V consumption, supporting the “five a day” advice [17].

A systematic review carried out by the advisory committee for the 2020 update of the US dietary guidelines, conducted on 1 randomized clinical trial and 152 observational studies, involving adults and elderly people from countries on different continents, demonstrated that diets rich in vegetables, fruit, legumes, nuts, whole grains, unsaturated vegetable oils, fish, and lean meat or poultry, were linked to a reduced risk of all-cause mortality. Moreover, these dietary patterns were characterized by relatively low levels of intake of red and processed meats, high-fat dairy products, and refined or sweet carbohydrates [12]. This evidence also suggested that the evaluation of the effects associated with specific food groups, such as F&V, should take into account that often such foods can represent only part of more complex dietary patterns, which as a whole are healthy [3].

A meta-analysis performed on 11 prospective studies (350,452 participants) linked dietary protein consumption with general and cause-specific mortality, highlighting that a high total protein intake is linked to a high all-cause mortality (RR for highest vs. lowest quantile: 1.05; CI: 1.01–1.10). This finding is mainly explained by a higher intake of animal proteins (deriving from meat and dairy products) associated with cardiovascular disease mortality. According to the analysis of the collected data, the authors found that the median animal protein intake corresponding to the highest and lowest quintile were about 75 g/day and 38 g/day, respectively, suggesting that a reduction in animal protein intake from 75 g/day to 42 g/day could lower the risk of CVD mortality of about 9%. On the other hand, a high intake of plant protein was linked to lower all-cause and CVD mortality (RR for all-cause mortality: 0.93; CI: 0.87–0.99) [16].

A systematic review and meta-analysis conducted on 69 prospective studies highlighted that a greater intake and/or blood concentrations of vitamins (E and C) and carotenoids were linked to a reduced risk of all-cause mortality (RR for dietary vitamin C intake: 0.89; 95% CI: 0.85–0.94; RR for blood concentrations of vitamin C: 0.72; 95% CI: 0.66–0.79) and also of cardiovascular disease and cancer. These findings support the recommendation to increase F&V intake for chronic disease prevention, as opposed to the use of antioxidant supplements [15]. Indeed, randomized clinical trials evaluating the correlation between antioxidant supplements intake and chronic disease prevention revealed no clear beneficial effects; therefore, it would be preferable to obtain antioxidants from natural dietary sources, which also contain a multitude of other bioactive substances, potentially playing a synergistic role.

An umbrella review of observational studies highlighted that the strongest evidence of protective effect was observed in relation to cardiovascular diseases, while evidence of decreased risk of all-cause mortality, along with other diseases, was rather limited. This was explained by the significant heterogeneity of results between studies, along with several confounding factors that limited the strength of the evidence. Indeed, the authors suggest that further studies are necessary to clarify the role of potential confounding factors and to hypothesize causation [13].

An updated meta-analysis of approximately 30 prospective cohort studies has also confirmed the inverse association of adherence to the Mediterranean diet with all-cause mortality. In particular, it was reported that SRR (Summary Relative Risks) for the study-specific highest/lowest, and per 1SD (standard deviation) MDS (Mediterranean Diet Score) increments, were 0.79 (95% CI: 0.77–0.81) and 0.92 (95% CI: 0.90–0.94), respectively. Moreover, it was highlighted that F&V were among the dietary components mostly involved in this association (fruit: 0.88, 95% CI: 0.83–0.94; vegetables: 0.94, 95% CI: 0.89–0.98) [14].

It is therefore possible to conclude that, taken together, the most recent studies have confirmed that a greater consumption of F&V is associated with a reduced risk of all-cause mortality and, in particular, with a reduced risk of mortality from cardiovascular diseases.

## 4. Effects of Fruit and Vegetable Consumption on Cancer

Worldwide, it has been calculated that in 2019 there were 23.6 million new cases of cancer (17.2 million when excluding nonmelanoma skin cancer) and nearly 10 million deaths in 2020, with a predicted 250 million DALYs attributable to cancer [42]. Based on such estimates, it is deduced that compared to 2010, there is a rise of 26.3% in new cases, 20.9% in deaths, and lastly 16.0% in DALYs. According to the number of deaths, years of life lost, and DALYs reported by the GBD 2019 study, cancer is the second major illness after cardiovascular diseases.

The selected six meta-analyses and systematic reviews focused on cancer included in the present review (Table 1) are described and discussed in detail below. In the meta-analysis of 26 cohort studies by Wang and Colleagues, a nonlinear inverse association (*P_nonlinear_* < 0.001) of F&V intake with overall mortality attributable to cancer was observed [17]. The dose–response meta-analysis demonstrated that, as compared with the reference F&V intake value (two servings/d), five daily servings of F&V were related to a reduced risk of cancer mortality (HR: 0.90; (0.86–0.95)). Moreover, such intake value was associated with the lowest risk of mortality, with any further decrease above that level. The authors also emphasized that the results of this new study supported a protective effect against cancer mortality exerted by a high intake of fruit, but not vegetables, whereas the World Cancer Research Fund/American Institute for Cancer Research previously reported that the correlation between high intakes of F&V and decreased risk of cancer mortality was either probable or limited [43]. As a possible explanation for this discrepancy, the authors argued that the duration of the follow-up period considered in previous studies was not sufficiently extended to detect the protective effect, given the long time that it often takes the development of cancer, even up to several decades.

In Europe, the European Prospective Investigation into Cancer and Nutrition (EPIC), one of the largest multicentric prospective cohort studies globally (519,978 participants, aged 35–70 years, recruited between 1992 and 1998 in 10 different European countries), was set up to provide robust evidence to investigate the relationship between diet and cancer (https://epic.iarc.fr (accessed on 23 June 2024)). In 2021, a systematic review of 110 studies was published to collect and analyze the results of the EPIC cohort concerning the relationship between diet exposure and cancer risk and mortality, specifically focusing on breast, colorectal, lung, and prostate cancer, as the most frequent types of neoplasies in the European population [21].

### 4.1. Colon Cancer

From two studies investigating the relationship between F&V consumption and colon cancer risk, it emerged that higher consumption (highest quintile vs. lowest) was associated with a lower risk, with similar HRs of 0.86 (0.75–1.00) and 0.87 (0.75–1.01), but this result was not observed when fruit consumption was evaluated separately from vegetable consumption. Other studies have highlighted a similar association between fiber consumption and reduced colorectal cancer risk, with HRs ranging from 0.58 (0.41–0.85) to 0.87 (0.79–0.96) per 10 g/day increase in fiber.

### 4.2. Breast Cancer

As regards breast cancer, low consumption of F&V has been observed to be associated with a higher risk of breast cancer (HR: 1.76 (1.10–2.82). In other distinct studies, vegetable consumption (highest quintile vs. lowest) was associated with a lower risk, with HRs ranging from 0.65 (0.53–0.81) to 0.87 (0.80–0.94). Additionally, protective effects associated with different types of vegetables were found for leafy vegetables (HR: 0.70 (0.57–0.86)), fruiting vegetables (HR: 0.75 (0.60–0.94)), and raw tomatoes (HR: 0.82 (0.66–1.01)). On the contrary, one study showed no associations between F&V intake and breast cancer risk [21]. In a distinct systematic review and meta-analysis of 25 prospective studies on pre- and post-menopausal women, Farvid et al. (2021) provided evidence that high total F&V consumption was associated with reduced risk of overall, postmenopausal, estrogen- and progesterone-receptor-positive and -negative breast cancer [22]. Total F&V consumption was associated with lower overall (RR: 0.91; 95% CI: 0.87–0.95) and postmenopausal breast cancer risk (RR: 0.88; 95% CI: 0.79–0.99). In particular, total fruit consumption was associated with lower overall (RR: 0.93; 95% CI: 0.88–0.99) and postmenopausal breast cancer risk (RR: 0.93; 95% CI: 0.87–0.99). Conversely, a high intake of fruit juices may increase the risk of breast cancer (RR: 1.04; 95% CI: 1.01–1.07), consistent with guidelines that distinguish between the relative benefits of whole fruit intake versus fruit-based beverages.

### 4.3. Lung Cancer

Results concerning lung cancer from the EPIC cohort derive from a limited number of studies, three of which focus on the effect of F&V consumption and analyzed by Ubago-Guisado et al. (2021) [21]. Overall, results suggest that the combined consumption of F&V was linked with a lower risk of lung cancer (for each 100 g/day increase in consumption), with an HR of 0.94 (0.89–0.99). Lung cancer risk decreased with high fruit consumption (highest quintile vs. lowest), with HRs ranging from 0.60 (0.46–0.78) to 0.75 (0.49–0.96), as well as with increased vegetable consumption (for each 100 g/day increase), with HRs ranging from 0.92 (0.85–0.99) to 0.94 (0.83–1.07). The basis for the 100 g/day increase was defined using the lowest quintile as the reference category (112.8 g/day for fruit and 124.2 g/day for vegetables), as reported by Buchner et al. (2010) [44]. Conversely, two studies showed no associations between vegetable intake and lung cancer [21]. Therefore, the protective role of vegetable consumption with respect to lung cancer remains to be clarified.

Another meta-analysis of prospective cohort studies investigated the relationships between F&V intake and lung cancer risk among participants with different smoking statuses [19]. Dose–response analysis indicated lung cancer risk was reduced by 5% (95%, CI: 0.93–0.97) and by 4% (95%, CI: 0.93–0.98) in current and former smokers with an increase of 100 g of fruit intake per day, respectively. Moreover, a 3% reduction in lung cancer risk (95%, CI: 0.96–1.00) in current smokers with an increase of 100 g per day of vegetable intake was observed.

### 4.4. Prostate Cancer

In the two studies on F&V consumption and prostate cancer from the EPIC study, no association between consumption of F&V and prostate cancer risk was observed in one study, whereas a protective association (HR:0.91 (0.83–0.99)) with the consumption of fruits (highest vs. lowest quintile) was observed in the other study [21].

As a whole, the studies from the EPIC cohort point to a protective effect of F&V consumption towards lung, breast, and colorectal cancer, whereas for prostate cancer, a protective effect was exerted only by fruit consumption.

### 4.5. Other Types of Cancer

A systematic review summarized published meta-analyses on the global burden of diseases attributable to low F&V consumption and the best estimates of relative risk. The review included 64 reports investigating 98 risk-disease pairs. Regarding tumors, the highest linear dose–response identified as protective for each 100 g/day increase in fruit intake was highlighted for esophageal cancer, followed by oral, pharyngeal, and laryngeal cancer, while the highest linear dose–response identified for each 100 g/day increase in vegetable intake was highlighted for renal carcinoma, followed by non-Hodgkin lymphoma [18].

The literature on F&V consumption is not only focused on assessing the risk of developing the disease but also on evaluating the risk of cancer recurrence, mortality, and all-cause mortality in cancer patients. In a 2020 systematic review and meta-analysis analyzing 28 cohort studies involving 18,278 males and females, aged 16–84, from Europe, North America, Asia, and Australia, with follow-up periods ranging from 9.1 to 16 years, the authors found that high vegetable intake preceding diagnosis was inversely associated with overall mortality in patients surviving from head and neck (HR: 0.75; 95% CI: 0.65–0.87) or ovarian cancer (HR: 0.78; 95% CI: 0.66–0.91) [20]. Furthermore, in subjects affected by ovarian cancer, fruit intake preceding diagnosis was also inversely correlated with all-cause mortality (HR: 0.82; 95% CI: 0.70–0.96). According to the authors, the general recommendation of five or more daily F&V servings (corresponding to 400 g/day) may partly underestimate the adequate requirements for cancer survivors, at least regarding ovarian cancer, suggesting an increased intake of approximately 600 g/day (i.e., 300 g/day of vegetables and 300 g/day of fruits).

By summarizing the evidence from recent studies on the relationship between F&V and cancer, it can be concluded that while there is general consensus on the beneficial effects linked to the consumption of F&V regarding cancer risk, there is no equivalent consensus on the extent of sustained effects on cancer risk over time [3,17,21,43].

## 5. Effects of Fruit and Vegetable Consumption on Cardiovascular Diseases

Cardiovascular diseases (CVDs) are the most common cause of death in the world, as estimated by the Global Burden of Disease (GBD) study, which reported 17.8 million CVD-related deaths worldwide in 2017, which represents a 21% increase with respect to the past decade. Among CVDs, ischemic heart disease and stroke account for approximately 50% and 35% of these deaths, respectively. The same situation is observed in Europe, where CVDs (particularly ischemic heart disease and stroke) cause almost 4 million deaths every year, accounting for around 44% of all deaths [45].

The protective and preventive effect of F&V consumption against CVDs has been demonstrated by robust scientific evidence deriving from epidemiological, observational, and intervention studies, recently analyzed by an exhaustive umbrella review [3].

The description and discussion of the nine meta-analyses and systematic reviews relating to the category of CVD pathologies selected and included in the present review (Table 1) are provided below.

There is a huge body of scientific evidence on diet and cardiovascular mortality. However, a restricted number of studies evaluated the effects of long-term consumption of food groups on cardiovascular health. A recent meta-analysis and systematic review aimed at analyzing the correlation between long-term consumption (considering a period greater than 5 years) of the main food groups (represented by whole grains, vegetables, fruit, nuts, legumes, processed meat, poultry, eggs, dairy products, and seafood) and cardiovascular mortality. The adopted strategy involved a dose–response analysis applying stringent selection criteria in order to include only cohort studies that presented repeated measures of dietary intake during the observation period. The work was conducted by analyzing 22 prospective studies on the general population from Europe, the United Kingdom, the USA, Australia, and Asia. In particular, the evaluation of the hazard ratio (HR) highlighted that long-term consumption of F&V (HR: 0.72; 95% CI: 0.61–0.85; *p* < 0.0001) and nuts (HR: 0.73; 95% CI: 0.66–0.81; *p* < 0.00001) significantly reduced cardiovascular mortality [26].

It is known that hypertriglyceridemia represents an important risk factor for CVD [46], and a high consumption of F&V is recommended for cardiovascular health. However, there is a persistent belief that fruit intake, due to high fructose content, increases the hematic level of triglycerides, unlike vegetables.

A systematic review and meta-analysis, aimed at clarifying the relationship between fruit intake and hypertriglyceridemia, conducted by analyzing five observational and two intervention studies on healthy or obese European, American, and Asian subjects, or those with a history of coronary heart disease, has highlighted that a high consumption of fruit but not vegetables is inversely correlated to hypertriglyceridemia [29]. This means that, in the context of triglyceride level control, it could be preferable to consider high fruit consumption separately from vegetable consumption. However, the authors highlight some weaknesses in the analysis conducted, due to the scarce number of eligible studies, especially the intervention ones. It is therefore recommended to perform further studies to clarify whether increased fruit intake can reduce the development of hypertriglyceridemia. Furthermore, more in-depth investigations are needed to establish the mechanisms that mediate the protective effects of fruit against hypertriglyceridemia and to specifically identify the fruit subtypes implicated in this action.

Two publications addressed the topic of heart failure and/or stroke. The first focused on the relationship between 10 food groups and the risk of developing coronary heart disease, heart failure, and stroke, applying a meta-analysis to 123 prospective studies, 32 of which related to vegetables, 30 to fruit, and 12 to nuts, conducted on the general European, American, and Asian healthy population [28]. The results showed that a high intake of vegetables, fresh fruit, and nuts lowers the risk of coronary heart disease (CHD), stroke, and heart failure. In the linear dose–response meta-analysis, there was in fact an inverse association, as evidenced by the Risk Ratios (RR) values, for separate consumption of vegetables and fruit (RR_CHD_: 0.97 (95% CI: 0.96–0.99), 0.94 (95% CI: 0.90–0.97), respectively; RR_stroke_: 0.92 (95% CI: 0.86–0.98), 0.90 (95% CI: 0.84–0.97)) and nuts (RR_CHD_: 0.67 (95% CI: 0.43–1.05)). The estimation of summary RR and 95% CI were conducted through a random effects model for highest versus lowest intake categories, and for linear and nonlinear relationships. According to the authors, the overall results of this meta-analysis can lay the foundation to derive specific dietary guidelines aimed at preventing CVD.

A second systematic review aimed at evaluating the effect of diet on the risk of stroke. The authors analyzed 12 observational and 16 intervention studies on healthy and diseased populations distributed across various continents (Europe, the United Kingdom, the USA, Asia, and Australia), concluding that adopting a diet rich in F&V in infancy can reduce the risk of stroke, compared to a diet based on meat and fat [25].

Hypertension is a known risk factor for several CVDs and is responsible for about 60% and 50% of strokes and coronary heart disease each year, respectively [47].

The systematic review and meta-analysis published by Madsen et al. (2023) analyzed 18 prospective studies on healthy populations affected by hypertension and distributed throughout the world (Europe, the USA, Asia, and Australia), highlighting that a high intake of F&V and total fruit, but not total vegetables, were linked to a lower risk of hypertension, which supports the recommendation to augment F&V ingestion as part of programs aimed at preventing hypertension. Notably, the correlation resulted in linear up to a daily intake of 800 g for F&V. In contrast, results for F&V subtypes were conflicting, suggesting the need to perform additional cohort studies in order to clarify this aspect [27].

Other authors conducted a systematic review and meta-analysis to verify the effect of dietary interventions on blood pressure in obese or overweight subjects. Ten intervention studies were included in the meta-analysis, showing that increased F&V consumption reduced systolic and diastolic blood pressure in obese and overweight individuals, which may reduce the risk of cardiovascular events [30].

Although the results appeared encouraging, the authors suggest interpreting them with caution, recognizing as the main weakness the scarce number of studies fulfilling the inclusion criteria set for eligibility and recommending an increased quality of future studies, which should also focus on the effect of interventions in defined subgroups of the population.

A systematic review carried out on 19 publications related to observational and intervention studies, focused on the relationship between lifestyle and cardio- and cerebrovascular diseases in middle-aged subjects, showed that high adherence to diets rich in plant-based foods, including components such as fruit, vegetables, nuts, whole grains, legumes, low-fat dairy products, olive oil, and a low intake of sodium, sugary drinks, alcohol, and red and processed meats, results in a lower risk of developing cardiovascular and cerebrovascular diseases in a dose-dependent manner [31]. As a general consideration, less meat and animal-derived food consumption appears to be beneficial for cardiovascular prevention. In particular, the Mediterranean diet and the DASH diet were acknowledged by the authors to have a more robust protective role for CVD.

Cardiorespiratory fitness (CRF) is the capacity of the circulatory and respiratory systems to provide oxygen to skeletal muscles during intense physical activity. This parameter allows the evaluation of the functional capacity of the respiratory and cardiovascular systems. Compared to traditional CVD risk factors, little is known about the interaction between diet and CRF. A systematic review carried out on 11 observational studies conducted on a wide range of populations from different countries and regions (Europe, the USA, Asia, New Zealand, and South America) showed that despite some inconsistent results, there is an overall positive correlation between dietary habits based on high levels of F&V consumption, as well as closer adherence to high-quality diets, in particular the Mediterranean diet, with higher CRF values [24].

Finally, a meta-analysis conducted on 66 randomized studies (for a total of 3595 participants) evaluated the effects of 10 food groups on intermediate disease markers, providing evidence that F&V were most effective in reducing systolic blood pressure, while nuts, legumes, and whole grains were ranked best at improving metabolic health (in terms of reduction in LDL cholesterol and, limited to nuts, of triglycerides) as compared with other food groups [23].

Overall, the analysis of the above-mentioned studies performed in the present review supports the evidence that F&V consumption exerts a strong effect in the prevention of CVDs.

## 6. Effects of Fruit and Vegetable Consumption on Obesity and Weight Loss

Although previous WHO/FAO reports have suggested a significant role for increased consumption of F&V in weight management, only a limited number of studies have specifically analyzed the relationship between F&V consumption and weight loss [3]. While a substantial body of short-term intervention studies exists regarding the effects of diet on weight reduction in obese patients, there is less evidence regarding the role of specific food groups and their optimal intake levels in obesity prevention. In the present review, a single comprehensive systematic review, accompanied by a meta-analysis, investigating the relationship between F&V consumption and obesity was selected (Table 1). This study examined prospective observational studies on the association between the intake of 12 food groups and the risk of weight gain, abdominal obesity, and overweight/obesity [32]. The study reviewed 43 reports from 25 prospective studies, of which 7, 6, and 4 were included in the meta-analysis related to vegetable, fruit, and nut consumption, respectively. Overall, the results showed that high levels of F&V intake were associated with a reduced risk of overweight/obesity, abdominal obesity, and weight gain. Specifically, the dose–response meta-analysis revealed an inverse association between fruit intake and the risk of overweight/obesity (RR: 0.93; 95% CI: 0.86, 1.00) and weight gain (RR: 0.91; 95% CI: 0.86, 0.97). Moreover, consumption of nuts was inversely associated with the risk of abdominal obesity (RR: 0.42; 95% CI: 0.31, 0.57). Although the results align with public health recommendations for a healthy diet, the authors considered the evidence collected of “very low to low” quality, suggesting caution in interpreting the results and recommending future studies with more appropriately designed observational studies and additional evidence from intervention studies. Other authors have recently observed in an umbrella review, which is, as such, not included in our selection of systematic reviews reported in Table 1, that observational studies and limited clinical trials do not produce convincing proof of the effectiveness of recommendations of consuming more F&V for weight reduction without a concurrent explicit commitment to reduce other sources of energy [3]. In essence, it is considered unlikely that promoting an increase in F&V consumption, not accompanied by specific indications to reduce the consumption of high-energy-density foods, can lead to weight loss or play a role in weight maintenance per se.

## 7. Effects of Fruit and Vegetable Consumption on Other Pathologies

In recent years, many systematic reviews, accompanied by meta-analyses, have investigated the impact of F&V consumption on diseases other than cancer and cardiovascular diseases. The eight meta-analyses and systematic reviews selected from the research conducted in the present review and focused on these other diseases (Table 1) are described and discussed in detail below.

Regarding the effects of F&V consumption on the risk of developing type 2 diabetes mellitus (T2DM), a recent systematic review, accompanied by dose–response meta-analysis [33], updated previous analyses of the same type [48], including 16 additional prospective cohort studies published after the extensive meta-analysis in 2017. This new analysis included 23 cohort studies and confirmed previous meta-analyses, indicating that F&V consumption was weakly inversely associated with the onset of T2DM. The calculated summary RR for high vs. low consumption and for daily consumption of 200 g was 0.93 (95% CI: 0.89–0.98) and 0.98 (95% CI: 0.95–1.01) for combined F&V. Moreover, considered separately, the RR was 0.93 (95% CI: 0.90–0.97) and 0.96 (95% CI: 0.92–1.00) for fruit, and 0.95 (95% CI: 0.88–1.02) and 0.97 (95% CI: 0.94–1.01) for vegetables. Based on these studies, a reduced (7%) risk of T2DM was estimated for high F&V consumption compared to low consumption. Moreover, the consumption of raisins, grapes, grapefruit, blueberries, and apples appears to be linked to a reduction in the risk of T2DM, while the consumption of cantaloupe, potatoes, certain types of cruciferous vegetables (Brussels sprouts and cauliflower), fruit drinks and fruit juices seems to be associated with a higher risk. Nevertheless, these results are based on a limited number of observations and require further confirmation before drawing consolidated conclusions. Overall, the results of this meta-analysis support current advice to consume more F&V for T2DM prevention and suggest that certain types of F&V could have a particularly protective effect, while others may increase the risk.

A recent area of interest is focused on the effects of F&V consumption on chronic inflammatory bowel diseases (IBD). This group of conditions, which include Crohn’s disease and ulcerative colitis, have a multifactorial origin that is still only partially understood, while alterations of the gut microbiota are believed to play a significant role in their development. However, little is currently known about the influence of dietary factors on these diseases. On this topic, a systematic review, accompanied by meta-analysis, has been recently published [38], being based, for the first time, on prospective cohort studies rather than results from case–control studies as in previous studies. It examined the results from 11 studies on the link between fruit, vegetable, and fiber intake and the risk of IBD. The meta-analysis highlighted that both fruit and vegetable intake were significantly associated with the risk of ulcerative colitis (for fruit RR: 0.69, 95% CI: 0.55–0.86; for vegetables RR: 0.56, 95% CI: 0.48–0.66) and Crohn’s disease (for fruit RR: 0.47; 95% CI: 0.38–0.58; for vegetables RR: 0.52, 95% CI: 0.46–0.59). Additionally, a significant association was observed between fiber intake and a reduced risk of Crohn’s disease, whereas a link was not found for ulcerative colitis. Overall, the available data indicate a significant inverse association between F&V consumption and the risk of IBD and its subtypes, although the limited evidence suggests the need for future prospective and clinical studies to consolidate these findings.

A lot of studies have recently investigated the effects of F&V consumption on low-grade chronic inflammation, which is believed to play a role in the development of numerous non-communicable chronic diseases such as cancer, cardiovascular diseases, atherosclerosis, neurodegenerative diseases, osteoporosis, metabolic syndrome, type 2 diabetes, and others [3]. A recent systematic review, accompanied by meta-analysis, examined the association between a posteriori dietary patterns and low-grade chronic inflammation in healthy adults, assessed by determining the blood concentration of biomarkers of inflammatory status, such as C-reactive protein, adiponectin, and leptin [34]. Consistent with previous findings, the results of this review indicated a modest inverse association between healthy dietary patterns and inflammation, while a modest direct association emerges with Western dietary patterns. Moreover, high intake of F&V, along with reduced consumption of red and processed meats, refined grains, and sugary beverages, were common components of “reduced rank regression” (RRR)-derived dietary patterns and inversely associated with the inflammatory status. A systematic review of the literature was dedicated to examining the same association in adolescents and children (aged 2 to 19 years) [35]. The results of this review, which evaluated data from 53 publications, showed that the Mediterranean diet was inversely associated with the level of pro-inflammatory biomarkers, such as tumor necrosis factor-alpha, interleukin-6, and C-reactive protein; in addition, these markers were also inversely associated with the intake of F&V, fiber, and vitamins C, A, and E, although the need for further studies to confirm this finding was emphasized.

An increasing number of studies have been dedicated to the effects of F&V consumption on bone health and the potential protective effect against osteoporosis. A systematic review, with meta-analysis, of 18 observational studies involving a total of 12,543 women highlighted that higher F&V consumption was associated with a lowered risk of postmenopausal osteoporosis, with a reduction of 32% and 13%, respectively [37]. Another recent systematic review of the literature, with meta-analysis, examined 13 publications related to 6 cohort studies (covering a population of 225,062 subjects) and 4 randomized controlled clinical trials, revealing that a diet rich in F&V was associated with a decreased risk of bone fractures [39]. In particular, the meta-analysis of cohort studies showed a reduction in the risk of hip fracture (HR: 0.92; 95% CI: 0.87–0.90) and overall fracture risk (HR: 0.90; 95% CI: 0.86–0.96) associated with regular F&V consumption in both men and women. The results pointed to moderate-quality evidence of a reduced risk of fractures linked to an increase in daily consumption of F&V.

As regards respiratory diseases, in the above-reported meta-analysis of 26 cohort studies, a nonlinear inverse association (*P_nonlinear_* < 0.001) of F&V intake with cause-specific mortality attributable to respiratory disease was also observed [17]. It was also estimated that the consumption of five servings of F&V per day, when compared with the reference intake level of two servings per day, was associated with a reduced risk for respiratory disease mortality, with an HR of 0.65 (0.59–0.72). Again, the consumption of three servings of vegetables and two servings of fruit daily was associated with the lowest risks of respiratory disease mortality. Moreover, a systematic review of eight observational studies examined the relationship between intake of F&V risk of chronic obstructive pulmonary disease (COPD) [36]. The meta-analysis included results from 5787 clinical cases within a population of 244,154 subjects and highlighted a 25% reduction in COPD risk when comparing the highest and lowest levels of F&V consumption, with similar reductions in the data disaggregated for fruit (28%) or vegetable (24%) consumption, respectively. As with most meta-analyses mentioned in this section, the need to consolidate the observed evidence through the implementation of larger cohort studies in different populations and the collection of consumption data using multiple detection methods was underlined.

## 8. Conclusions

As regards the benefits for human health, overall, the analysis of systematic reviews and meta-analysis conducted within the timeframe considered in this review confirmed and consolidated the evidence gathered in recent decades regarding the protective role of F&V consumption against the development of NCDs. In particular, the strongest evidence was demonstrated for CVDs. However, a general consideration emerging from our analysis is the urgent need to perform further studies, particularly randomized control trials, aimed at corroborating the existing evidence with robust results and at clarifying the role of confounding factors that often hamper a correct interpretation of the outcomes. Designing an RCT to evaluate the health effects of fruit and vegetable consumption should involve several key steps, including the following: selection of the target population, according to defined inclusion and exclusion criteria; estimation of the number of participants needed to detect a statistically significant difference between groups; definition of the duration of the intervention based on the expected timeline for observing changes (short or long term); monitoring the adherence to the dietary intervention through dietary recalls, food diaries, or biomarker assessments; and adjusting for confounding factors such as baseline health status, age, gender, and physical activity levels.

Moreover, applying more standardized approaches and study designs could help in improving the comparative analysis of different studies. To this respect, a recent tool for assessing the quality of evidence for risk–outcome associations in an objective and quantitative manner has been designed to aid in overcoming methodological issues [49]. Finally, the availability of more accurate and detailed food intake data would be helpful to obtain stronger evidence on the relationship between diet and health outcomes, as well as to support nutritional education programs and public health policies aimed at increasing F&V consumption. Applying research results on the health effects of fruit and vegetable consumption to public health policies should envisage a systematic process that bridges scientific findings with policy-making, aiming at improving population-level health outcomes. Specifically, translating research findings into actionable public health policies should rely on combining results from RCTs, cohort studies, and meta-analyses to clearly demonstrate the health benefits of F&V; providing concrete data on how increased F&V consumption impacts specific health indicators; identifying specific population subgroups that benefit most; and aligning the evidence with national and international health goals.

An aspect that is also worth considering is that joining the two categories of fruits and vegetables into one could be misleading, given their different roles in a healthy diet, as well as their level of consumption that varies among dietary habits [50]. However, these food categories are often referred to as pooled. In the present work, aimed at collecting and analyzing the most recent systematic reviews and meta-analyses in the field, the data were reported as they appeared in those studies. When data were reported for fruits and vegetables as separate categories, we maintained this distinction, while we referred to F&V when it was not possible to distinguish between them. However, there is a growing need to investigate separately the impact of fruit and vegetable consumption on chronic diseases. The results reported in this review indicate different trends depending on the type of pathology. Nevertheless, an emerging trend emphasizes the need to consume more vegetables compared to fruit. For example, in the study by Wang et al. (2021) [17], the thresholds for risk reduction in mortality are identified as two servings daily for fruit intake and three servings daily for vegetable intake. The main factors supporting this trend include lower sugar content, higher nutrient density, higher fiber content, as well as greater satiety potential, among others. In addition, a growing body of evidence is being gathered, for example, regarding the protective effect of vegetable consumption against chronic diseases, such as ischemic heart disease (IHD), ischemic stroke, hemorrhagic stroke, type 2 diabetes, and esophageal cancer [51]. In light of this, further analysis of the protective role of fruit and vegetables as separate food categories appears to be one of the most interesting areas to investigate in the near future.

We recognize as a limitation of the present work its narrow originality. However, we believe that our analysis was useful in collecting and summarizing the most recent evidence in the field, and also highlighting existing issues and gaps, which could help in addressing future research to better establish a causal relationship between F&V consumption and human health.

## Figures and Tables

**Table 1:** List of selected systematic reviews and meta-analyses assessing fruit and vegetable intake in relation to disease types.

Type of Disease	Country	Target Population	Type and Number of Studies Included (Observational/Intervention)	Meta-Analysis Performed (Y/N)	Outcome	Reference
All-cause mortality	All continents	Adults, older	Observational (n = 152), Intervention (n = 1)	Y	Dietary patterns characterized by greater intake of F&V (among other food groups) are associated with a reduced risk of all-cause mortality.	[12]
All-cause mortality	All countries	General population	Observational (n = 60)	N	Rather limited evidence linking F&V consumption with decreased risk of all-cause mortality. Authors claim heterogeneity of results between studies and several confounding factors.	[13]
All-cause mortality	All countries	General population	Observational (n = 30)	Y	Inverse association of adherence to Mediterranean diet with all-cause mortality, with F&V mostly involved in this association.	[14]
All-cause mortality	EU, America, Asia	Adults	Observational (n = 60)	Y	Greater dietary intake and/or blood concentrations of markers of F&V intake associated with a reduced risk of all-cause mortality.	[15]
All-cause mortality	EU	Adults	Observational (n = 11)	Y	Higher plant protein intake associated with lower all-cause and CVD mortality.	[16]
All-cause mortality, cancer, other (respiratory diseases)	USA	Adults	Observational (n = 28)	Y	Robustly highlighted association between a greater intake of F&V with a lower risk of total and cause-specific mortality. Minimum intake to assure reduction in mortality risk: 2 portions/day (F) and 3 portions/day (V).	[17]
Cancer	All countries	General population	Observational (n = 64)	N	Within 98 risk-NCD pairs, the highest protective effect for each 100 g/day increase in fruit intake highlighted for esophageal cancer, followed by oral, pharyngeal, and laryngeal cancer; for 100 g/day increase in vegetable intake for renal carcinoma, followed by non-Hodgkin lymphoma.	[18]
Cancer	All countries	Smokers	Observational (n = 26)	Y	Risk of lung cancer reduced in current and former smokers with an increase in fruit intake, and in current smokers with an increase in vegetable intake.	[19]
Cancer	EU, America, Asia, Australia	General population	Observational (n = 28)	Y	High vegetable intake before diagnosis inversely associated with overall mortality in survivors of head and neck or ovarian cancer. Recommendations of F&V consumption for cancer survivors could be increased to approximately 600 g/day.	[20]
Cancer	EU	General population	Observational (n = 110)	N	F&V consumption had a protective effect against colorectal, breast, and lung cancer, whereas only fruit had a protective effect against prostate cancer.	[21]
Cancer	Not indicated	Adult women	Observational (n = 25)	Y	Total F&V consumption associated with lower overall and postmenopausal breast cancer risk. Total fruit consumption associated with lower overall and postmenopausal breast cancer risk.	[22]
CVD	All countries	Adults	Intervention (n = 66)	Y	Evidence that F&V are effective in reducing systolic blood pressure.	[23]
CVD	EU, USA, Asia, New Zealand, South America	General population	Observational (n = 11)	N	Overall positive association between dietary patterns characterized by high levels of F&V consumption with higher cardiorespiratory fitness values.	[24]
CVD	EU, UK, USA, Asia, Australia	Healthy and diseased	Observational (n = 12), Intervention (n = 16)	N	Adopting a diet rich in F&V in the early stages of life can reduce the risk of stroke.	[25]
CVD	EU, UK, USA, Australia, Asia	General population	Observational (n = 22)	Y	Robust demonstration that long-term consumption of F&V significantly reduced cardiovascular mortality.	[26]
CVD	EU, USA, Asia, Australia	Healthy and diseased (hypertension cases)	Observational (n = 18)	Y	High intake of F&V and total fruit, but not total vegetables, associated with a reduced risk of hypertension. Association appeared to be linear up to an intake of 800 g/day for F&V.	[27]
CVD	EU, USA, Asia	General population	Observational (n = 123)	Y	High intake of vegetables, fresh fruit and nuts lowers the risk of coronary heart disease, stroke and heart failure.	[28]
CVD	EU, USA, Asia	Healthy and diseased (obese; history of CHD)	Observational (n = 5), Intervention (n = 2)	Y	High consumption of fruit but not vegetables is inversely associated with hypertriglyceridemia. Authors highlight some weaknesses in the analysis conducted, due to the limited number of eligible studies.	[29]
CVD	Not indicated	Overweight, obese	Intervention (n = 10)	Y	Increased F&V consumption reduced systolic and diastolic blood pressure in obese and overweight individuals.	[30]
CVD	Not indicated	Not indicated	Observational (n = 170), Intervention (n = 64)	N	High adherence to plant-based diets, including F&V, results in a lower risk of developing cardiovascular and cerebrovascular diseases in a dose-dependent manner.	[31]
Obesity	Not indicated	General population	Observational (n = 25)	Y	High levels of F&V intake associated with a reduced risk of overweight/obesity, abdominal obesity, and weight gain. Evidence judged of very low to low quality.	[32]
Other (T2D)	All countries	General population	Observational (n = 23)	N	Support current recommendations to increase F&V consumption for T2DM prevention. Certain types of F&V may have a particularly protective effect, while others may increase the risk.	[33]
Other (inflammation)	All countries	Adults, general population	Observational (n = 12)	Y	A modest inverse association observed between healthy dietary patterns (high intake of F&V, along with reduced consumption of red and processed meats, sugary beverages, and refined grains) and inflammation.	[34]
Other (inflammation)	All countries	Children and adolescents aged 2–19 years	Observational (n = 45), Intervention (n = 8)	Y	The Mediterranean diet, as well as the intake of F&V, fiber, and vitamins C, A, and E, inversely related to the level of pro-inflammatory biomarkers.	[35]
Other (pulmonary disease)	Denmark, Sweden, UK, China, Japan, USA	Chronic obstructive pulmonary disease (COPD) patients	Observational (n = 8)	N	Higher F&V, total and disaggregated, consumption associated with a reduced risk of COPD. Need to consolidate the observed evidence through larger cohort studies and multiple detection methods of consumption data.	[36]
Other (osteoporosis)	China, Korea, India, Iran	Post-menopausal women	Observational (n = 18)	Y	Higher F&V consumption associated with a reduction in the risk of postmenopausal osteoporosis.	[37]
Other (IBD)	EU, USA, Australia	Adults	Observational (n = 11)	Y	A significant inverse association between F&V consumption and the risk of IBD and its subtypes (ulcerative colitis and Crohn’s disease). Limited evidence and need for future prospective and clinical studies to consolidate these findings.	[38]
Other (bone health)	EU, USA, Iran	Post-menopausal women	Observational (n = 6), Intervention (n = 4)	Y	A diet rich in F&V associated with a decreased risk of bone fractures (hip and overall fracture risk). Evidence judged of moderate quality.	[39]

## Data Availability

Not applicable.
